# Early in-hospital use of SGLT2i in heart failure patients with ischemic etiology

**DOI:** 10.1007/s10557-025-07714-0

**Published:** 2025-05-15

**Authors:** Paolo Severino, Andrea D’Amato, Vincenzo Myftari, Silvia Prosperi, Marco Valerio Mariani, Lorenzo Colombo, Rosanna Germanò, Stefanie Marek-Iannucci, Claudia Cestiè, Federico Ferranti, Camilla Segato, Matteo Aulicino, Domenico Filomena, Giovanna Manzi, Nicola Pierucci, Gianluca Di Pietro, Lucia Ilaria Birtolo, Silvia Papa, Francesco Ciciarello, Gennaro Sardella, Massimo Mancone, Roberto Badagliacca, Carmine Dario Vizza

**Affiliations:** Department of Clinical, Internal, Anesthesiology and Cardiovascular Sciences, Sapienza University of Rome, 00161 Rome, Italy

**Keywords:** Heart failure, SGLT2i, Ischemic heart disease, Acute coronary syndrome, Hospitalization, Cardiovascular mortality

## Abstract

**Purpose:**

SGLT2i role in the treatment of heart failure (HF) regardless of clinical presentation and left ventricular ejection fraction (LVEF) has been widely proven and real-world data regarding patients with HF and ischemic heart disease (IHD) and, in particular with recent acute coronary syndrome (ACS) and de novo HF, are lacking. We aim to evaluate the occurrence of the composite of cardiovascular death (CV)/ HF hospitalization (HFH), all-cause death, CV death and HFH at 6 months follow up, in patients with HF due to IHD as well as in recent ACS who introduced SGLT2i during the index hospitalization.

**Methods:**

The present is an observational, prospective, single center study, enrolling patients with a diagnosis of HF due to IHD as primary etiology. According to SGLT2i introduction timing, two groups were created: pre-discharge (G1) or post-discharge (G2) introduction. A sub-analysis in patients admitted due to ACS has been performed.

**Results:**

A total of 222 consecutive patients have been enrolled from April 2022 to April 2024 and were followed-up for a period of 6 months. At multivariate Cox regression analysis, statistically significant differences have been observed between the two groups in terms of the composite CV death/HFH (HR = 0.24; 95%CI [0.101–0.564]; *p* = 0.001), all-cause death (HR = 0.27; 95% CI [0.100–0.725]; *p* = 0.009), CV death (HR = 0.32; 95%CI [0.101–0.999] *p* = 0.045) and HFH (HR = 0.31; 95%CI [0.098–0.963]; *p* = 0.043). Patients with ACS treated with SGLT2i before discharge showed a reduced rate of CV death/HFH (log-rank *p* = 0.008), CV death (log-rank *p* = 0.015) and all-cause death (log-rank *p* = 0.005) compared to patients who were not treated with SGLT2i before discharge. In this subpopulation, no differences have been observed in terms of HFH (log-rank *p* = 0.155). Significant differences in term of CV death/HFH (log-rank *p* = 0.039) have been observed in de novo HF patients, but not in terms of the other study endpoints.

**Conclusions:**

The early in-hospital introduction of SGLT2i reduced the occurrence of the composite CV death/HFH, all-cause death, CV death and HFH in patients with ischemic cardiomyopathy. In the subgroup analysis of patients admitted due to ACS, the introduction of SGLT2i during the index hospitalization resulted in a significant reduction of the composite CV death/HFH, CV death and all-cause death, but not in HFH. The same therapeutic strategy resulted in reduced rate of CV death/HFH in the de novo HF subpopulation.

## Introduction

Heart failure (HF) is a worldwide socioeconomic problem, associated with a high burden of morbidity and mortality [1, 2]. The age-adjusted incidence of HF has been decreasing in industrialized countries due to improved management of cardiovascular (CV) diseases. However, the overall incidence continues to rise, primarily due to the aging population [1, 2]. According to recent data, the global prevalence of HF has increased over the past few decades, with approximately 64 million people affected worldwide, and this number is expected to continue growing as the population ages [1–3]. The most common cause of HF worldwide is represented by ischemic heart disease (IHD) [1–3]. IHD accounts for up to 70% of HF cases in developed countries. In particular, the occurrence of acute myocardial infarction (AMI) and all its sequelae, such as left ventricular dysfunction, myocardial scarring, fibrosis and remodeling, represent the pathophysiological link between IHD and HF [4]. Epidemiological data show that HF has a prevalence of about 50% in patients with a history of AMI within 5 years [1–4]. In addition, chronic coronary artery disease (CAD) is associated with increased incidence of HF, particularly when combined with advance age and CV risk factors [5]. Patients with HF with mildly reduced ejection fraction (HFmrEF) and HF with reduced ejection fraction (HFrEF) may have more underlying CAD compared to patients with HF with persevered ejection fraction (HFpEF), in which coronary microvascular dysfunction (CMD) represents the main pathophysiological substrate for IHD [1, 5].

The introduction of the four pillars of HF therapy revolutionized the HF patient’s prognosis, reducing mortality and rehospitalization due to HF and, therefore disease progression [1, 6–10]. The latest Guidelines update [10], and several studies [11–14] demonstrated the importance of the rapid introduction and up titration of disease modifying drugs. The benefit derived from the early initiation of SGLT2i in the course of HF, regardless of left ventricular ejection fraction (LVEF) and symptoms has been demonstrated [10]. Furthermore, when initiated early, they also improve short term outcomes in patients hospitalized due to acute HF, improving also congestion status, renal function and symptoms [15, 16]. The current Guidelines [2] lack evidence regarding the early use of SGLT2i in patients admitted due to acute coronary syndrome (ACS) and HF. The EMPACT-MI trial [17] did not show a reduced risk of all-cause mortality and HF hospitalization (HFH) in patients at risk for HF and recent ACS, and the DAPA-MI trial [18] showed an improvement in cardiometabolic outcome but not in major adverse events, while several other studies [19–23] reported conflicting results in this population.

The aim of this manuscript is to study the effect of early, in-hospital SGLT2i initiation focusing on patients with HF due to IHD, with particular regard to the subpopulation admitted due to ACS, in terms of the composite of CV death and HFH, all-cause death, as well as each single endpoint of the composite outcome.

## Methods

The present is an observational, prospective, single center study, enrolling patients hospitalized due to HF as primary diagnosis at the Cardiology and Intensive Care Unit divisions of the Department of Clinical, Internal, Anesthesiology and Cardiovascular Sciences at Policlinico Umberto I, Sapienza University of Rome. Inclusion criteria were the following: I) written, signed and dated informed consent; II) age above 18 years; III) diagnosis of HF due to IHD as primary etiology according to the Guidelines [2, 5]. Exclusion criteria were the following: I) any condition limiting life expectancy to less than one year; II) end-stage kidney failure and/or dialysis; III) planned or history of heart transplantation and ventricular assist device (VAD); IV) pregnancy or nursing; V) non-compliance with the study protocol; VI) prior use of SGLT2i.

Patients were divided in two groups according to SGLT2i introduction timing: G1 included patients who were treated with a SGLT2i during index hospitalization; G2 included patients who were treated SGLT2i subsequently.

A sub-analysis in patients admitted due to ACS has been performed to observe the effect of in-hospital SGLT2i introduction on main adverse outcomes. A further sub-analysis in patients with de novo HF has been performed.

The main reason for the difference in the timing of initiation between the two groups is related to the updated 2023 Guidelines for the diagnosis and treatment of acute and chronic HF [10], which recommend the use of SGLT2i across the entire LVEF spectrum, along with an intensive and rapid initiation prior to hospital discharge.

The following parameters have been collected: i) clinical parameters (past medical history, physical examination, electrocardiogram, arterial blood pressure, NYHA class, pharmacological therapy); ii) echocardiographic parameters (ventricular chambers size, systolic and diastolic function, valve disease and severity, tricuspid annular plane systolic excursion [TAPSE]); iii) laboratory parameters (blood cell count, creatinine, electrolytes, estimated glomerular filtration rate [eGFR]).

Over a follow-up period of 6 months after the index hospitalization, a composite of CV death/HFH, all-cause death, as well as CV death and HFH considered alone have been investigated in the outpatient HF clinic.

Data were anonymously collected in a dedicated Microsoft Excel Database. The study was conducted according to the Helsinki Declaration. The study protocol has been approved by the local Ethical Committee (rif.7068).

### Statistical Analysis

The normal distribution of continuous variables was assessed with the Kolmogorov–Smirnov test. Continuous variables were expressed as mean and standard deviation, whereas median and first and third quartiles were used for non-normally distributed data. Categorical data were described as numbers and percentage. Student’s t-test, Mann–Whitney test, the χ2 test, and the Fisher exact test were used for comparisons, as needed. The Kaplan–Meier method was used to estimate cumulative event rates of study outcomes in the overall population. Kaplan–Meier analysis was used to analyze differences in clinical outcome rates in the subgroup of patients with ACS and de novo HF. Differences in each group were compared using log-rank tests. The univariate and multivariate Cox regression analysis was performed to obtain hazard ratio (HR) of the association between in-hospital SGLT2i initiation and the endpoints. For all tests, a p-value < 0.05 was considered statistically significant.

The statistical analysis was performed using SPSS version 27.0 for Mac (IBM Software, Inc., Armonk, NY, USA).

## Results

A total of 222 consecutive patients have been enrolled from April 2022 to April 2024 and were followed-up for a period of 6 months post-discharge.

The median age of the study population was 73 years (± 15). 163 (73%) patients were males. 96 (43%) patients had a previous hospitalization due to HF. Mean NYHA class at admission was class III. Mean admission LVEF was 35% (± 17). 161 (72.5%) patients had HFrEF, 39 had HFmrEF (18%) and 22 (9.5%) had HFpEF. Mean TAPSE was 18 mm (± 6). Mean eGFR at admission was 63 ml/min (± 35). Baseline features of the patient population and discharge therapy have been listed in Table 1.

**Table 1:** Baseline features and discharge therapy of overall population and the two study groups

Variables	Overall population (*N* = 222)	G1 (*n* = 114)	G2 (*N* = 108)	*p*-value
Age (± SD)	73 (**± **15)	73 (**± **13)	73.5 (**± **15)	0.365
Male sex, n (%)	163 (73.4)	90 (78.9)	73 (67.6)	0.056
Previous HFH, n (%)	96 (43.2)	52 (45.6)	44 (40.7)	0.62
De novo HF, n (%)	126 (56.7)	62 (54.4)	64 (59.2)	0.499
SAH, n (%)	177 (79.7)	92 (80.7)	85 (78.7)	0.711
Type II diabetes mellitus, n (%)	84 (38)	46 (40.4)	38 (35.2)	0.428
Dyslipidemia, n (%)	152 (68)	83 (72.8)	69 (63.9)	0.153
Family history of CVD, n (%)	56 (25)	29 (25.4)	27 (25)	0.940
COPD, n (%)	53 (24)	24 (21.1)	29 (26.9)	0.311
Smoking habit, n (%)	116 (52)	63 (55.3)	53 (49.1)	0.356
ICD, n (%)	48 (21.6)	29 (25.4)	19 (17.6)	0.156
CRT-D, n (%)	12 (5.4)	7 (6.1)	5 (4.6)	0.619
PMK, n (%)	27 (12)	16 (14)	11 (10.2)	0.380
LVEF, % (± SD)	35 (**± **17)	31.5 (**± **16)	35 (**± **19.5)	0.145
TAPSE, mm (SD)	18 (6)	18 (6.2)	18.5 (5)	0.902
eGFR, ml/min (± SD)	63 (**± **35)	65 (**± **36)	61 (**± **39)	0.323
HFrEF, n (%)	161 (72.5)	85 (74.6)	76 (70.4)	0.484
HFmrEF, n (%)	39 (17.5)	20 (17.5)	19 (17.6)	0.992
HFpEF, n (%)	22 (10)	9 (7.9)	13 (12)	0.302
BB, n (%)	204 (92)	102 (89.5)	102 (94.4)	0.175
ACE-i/ARBs, n (%)	64 (29)	31 (27)	33 (30.5)	0.657
ARNI, n (%)	123 (55.4)	77 (67.5)	46 (42.6)	< 0.001
MRAs, n (%)	130 (58.5)	59 (79.7)	71 (76.3)	0.601
Loop diuretics, n (%)	155 (70)	77 (67.5)	78 (72.2)	0.448

*HFH* heart failure hospitalization, *SAH* systemic arterial hypertension, *CVD* cardiovascular disease, *COPD* chronic obstructive pulmonary disease, *ICD* implantable cardioverter defibrillator, *CRT-D* Cardiac resynchronization therapy with defibrillator, *PMK* pacemaker, *LVEF* left ventricular ejection fraction, *TAPSE* tricuspid annular plane systolic excursion, *eGFR* estimated glomerular filtration rate, *HFrEF* heart failure with reduced ejection fraction, *HFmrEF* heart failure with mildly reduced ejection fraction, *HFpEF* heart failure with preserved ejection fraction, *BB* beta blockers, *ACEi* angiotensin converting enzyme, *ARBs* angiotensin receptor blockers, *ARNI* angiotensin receptor/neprilysin inhibitor, *MRAs* mineralocorticoid receptor antagonists

Cox regression analysis showed statistically significant differences between the two groups in terms of the composite CV death/HFH (HR = 0.24; 95%CI [0.101–0.564]; *p* = 0.001), all-cause death (HR = 0.27; 95% CI [0.100–0.725]; *p* = 0.009), CV death (HR = 0.32; 95%CI [0.101–0.999] *p* = 0.045) and HFH (HR = 0.31; 95%CI [0.098–0.963]; *p* = 0.043) (Table 2).

**Table 2:** Occurrence of the adverse events in each group and multivariate Cox regression analysis for the composite outcome of CV death and HFH, all-cause death, as well as each of the single outcome composing the composite outcome

Outcomes	Overall population (*N* = 222)	G1 (*N *= 114)	G2 (*N* = 108)	HR (95%CI)	*p*-value
CV death/HFH, n (%)	28 (12.6)	7 (6.1)	21 (19.8)	0.24 (0.101–0.564)	0.001
CV death, n (%)	15 (6.75)	4 (3.5)	11 (10.4)	0.32 (0.101–0.999)	0.045
HFH, n (%)	15 (6.75)	4 (3.5)	11 (10.4)	0.31 (0.098–0.963)	0.043
All-cause death, n (%)	20 (9)	6 (5.2)	14 (12.9)	0.27 (0.100–0.725)	0.009

*CV *cardiovascular* HFH *heart failure hospitalization,* HR *hazard ratio*, CI confidence interval*

**Fig. 1:**
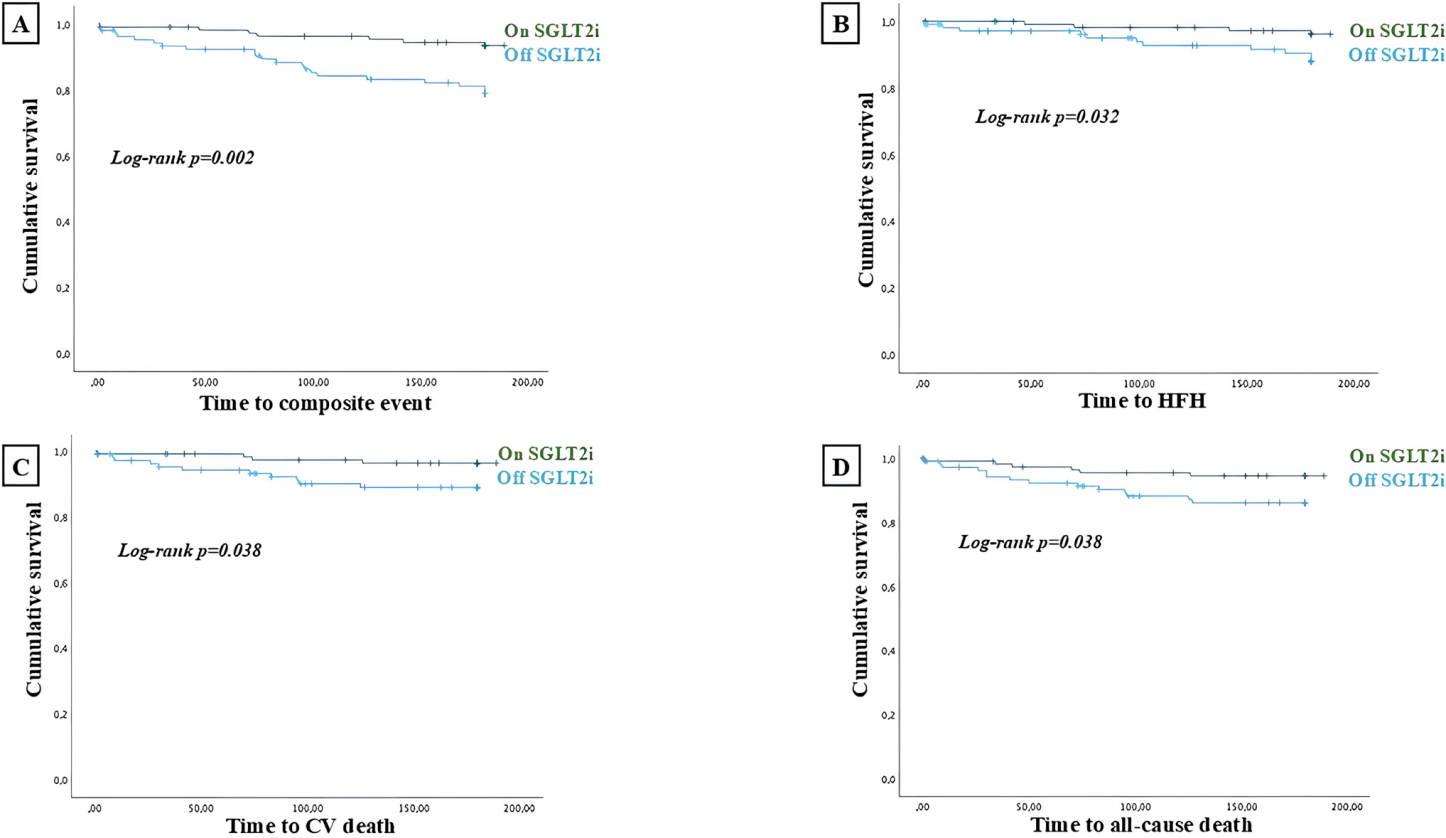
Survival analysis regarding the composite of CV death and HFH (**A**), each of the outcome of the composite endpoint in the two-study group (**B** and **C**) and all cause death (**D**), in the general population with ischemic cardiomyopathy. Time to the event has been expressed in days. “on SGLT2i” defines patients who introduced SGLT2i during hospitalization while “off SGLT2i” defines patients who did not start SGLT2i during hospitalization. *HFH: heart failure hospitalization; CV: cardiovascular. SGLT2i: sodium glucose cotransporter 2 inhibitors*

A Kaplan Meier survival analysis demonstrated that patients, who were started on a SGLT2i during, hospitalization, showed a significantly reduced rate of the composite CV death/HFH (log-rank *p* = 0.002), all-cause death (log-rank *p* = 0.038), CV death (log-rank *p* = 0.038) and HFH (log-rank *p* = 0.032) at 6 months follow up (Fig. 1).

**Fig. 2:**
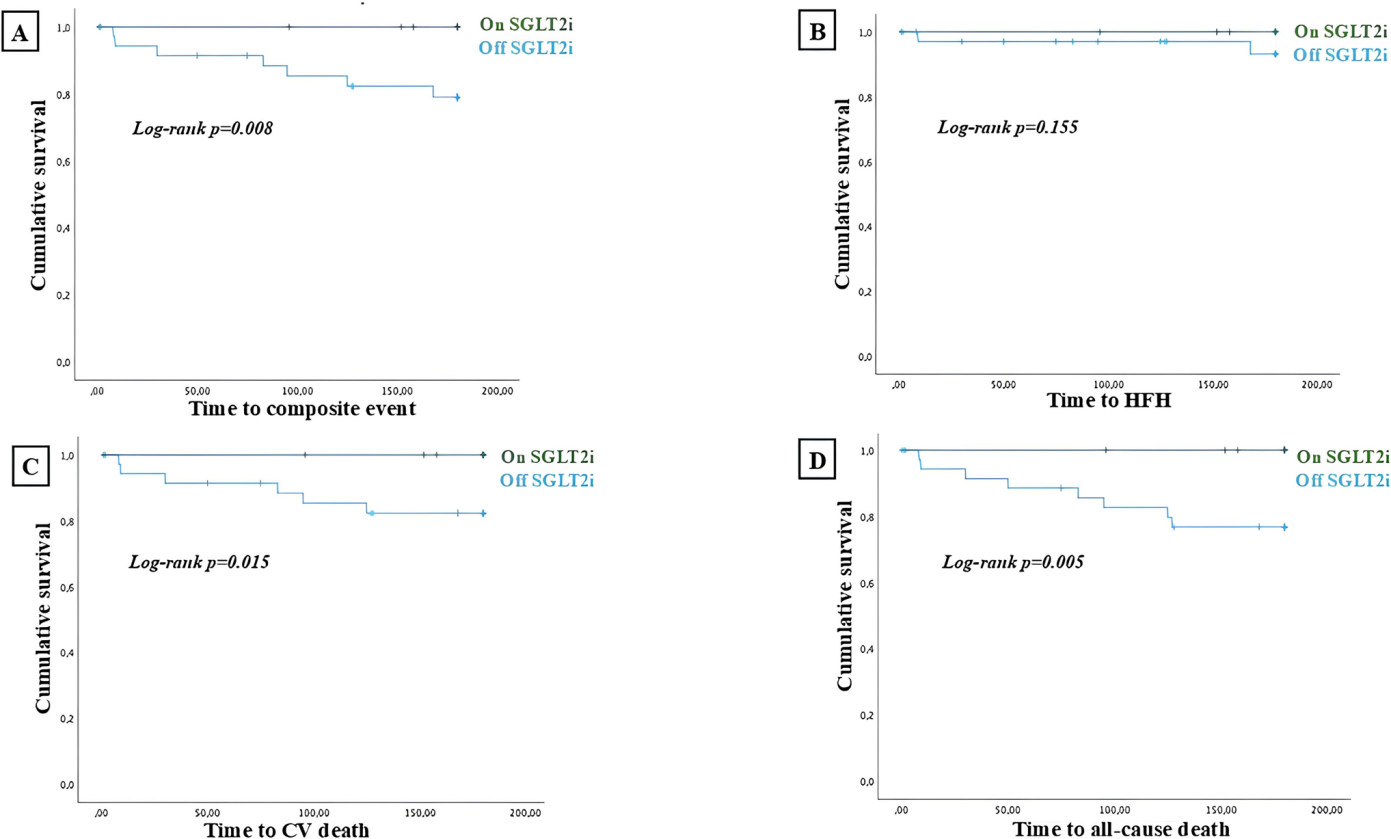
Survival analysis regarding the composite of CV death and HFH (**A**), each of the outcome of the composite endpoint (**B** and **C**) and all-cause death (**D**), in patients admitted due ACS and HF. Time to the event has been expressed in days. “on SGLT2i” defines patients who introduced SGLT2i during hospitalization while “off SGLT2i” defines patients who did not start SGLT2i during hospitalization *CV: cardiovascular; HFH: heart failure hospitalization; SGLT2i: sodium glucose cotransporter 2 inhibitors*

A subgroup analysis performed on patients admitted due to ACS and contextual HF has been performed. Baseline features and rate of adverse events of patients admitted due to ACS and HF have been listed in Tables 3 and 4 respectively. The Kaplan Meier survival analysis showed that patients with ACS treated with SGLT2i had a reduced rate of CV death/HFH (log-rank *p* = 0.008), all-cause death (log-rank *p* = 0.005), and CV death (log-rank *p* = 0.015) compared to patients not treated with SGLT2i before discharge. No differences have been observed in terms of HFH (log-rank *p* = 0.155) (Fig. 2).

**Table 3:** Baseline features, discharge therapy and rate of adverse events of the subpopulation admitted due to ACS and HF. Differences between patients who early started SGLT2i and patients who did not early start SGLT2i have been represented

Variables	ACS *(N* = 69)	SGLT2i on therapy (*N* = 32)	SGLT2i off therapy (*N *= 37)	*p*-value
Age (± SD)	76 (**± **17)	74.5 (**± **20)	77 (**± **11)	0.847
Male sex, n (%)	48 (69)	25 (78.1)	23 (62.2)	0.151
Previous HFH, n (%)	27 (39)	15 (46.9)	12 (32.4)	0.220
SAH, n (%)	57 (82.6)	27 (84.4)	30 (81.1)	0.719
Type II diabetes mellitus, n (%)	32 (46.3)	16 (50)	16 (43.2)	0.575
Dyslipidemia, n (%)	44 (63.7)	22 (68.8)	22 (59.5)	0.423
Family history of CVD, n (%)	9 (13)	5 (15.6)	4 (10.8)	0.554
COPD, n (%)	19 (27.5)	7 (21.9)	12 (32.4)	0.328
Smoking habit, n (%)	38 (55)	21 (65.5)	17 (45.9)	0.101
ICD, n (%)	5 (7.2)	3 (9.4)	2 (5.4)	0.657
CRT-D, n (%)	2 (3)	0 (0)	2 (5.4)	0.495
PMK, n (%)	9 (13.04)	3 (9.4)	6 (16.2)	0.489
LVEF, % (± SD)	35 (**± **12)	39 (**± **12)	35 (**± **12)	0.387
TAPSE, mm (SD)	17 (5)	19 (8)	17 (5)	0.008
eGFR, ml/min (± SD)	65 (**± **38)	70 (**± **35)	62.5 (**± **37)	0.068
HFrEF, n (%)	48 (69.6)	19 (59.4)	29 (78.4)	0.087
HFmrEF, n (%)	16 (23.2)	11 (34.4)	5 (13.5)	0.040
HFpEF, n (%)	5 (7.2)	2 (6.3)	3 (8.1)	0.739
BB, n (%)	63 (91.3)	29 (90.6)	34 (91.9)	0.852
ACE-i/ARBs, n (%)	19 (27.5)	8 (25)	11 (29.7)	0.788
ARNI, n (%)	38 (55)	21 (65.6)	17 (45.9)	0.101
MRAs, n (%)	43 (62.3)	17 (73.9)	26 (81.3)	0.516
Loop diuretics, n (%)	45 (65.2)	18 (56.3)	27 (73)	0.146
STEMI, n (%)	10 (14.5)	5 (15.6)	5 (13.5)	1
NSTEMI, n (%)	35 (50.5)	14 (43.7)	21 (56.7)	0.338
UA, n (%)	24 (35)	13 (40.7)	11 (29.8)	0.448
Complete revascularization	60 (87)	28 (87.5)	32 (86.4)	1
Medically managed ACS	9 (13)	4 (12.5)	5 (13.5)	1

*ACS* acute coronary syndrome, *SGLT2i* sodium glucose cotransporter 2 inhibitors, *HFH* heart failure hospitalization, *SAH* systemic arterial hypertension, *CVD* cardiovascular disease, *COPD* chronic obstructive pulmonary disease, *ICD* implantable cardioverter defibrillator, *CRT-D* Cardiac resynchronization therapy with defibrillator, *PMK* pacemaker, *LVEF* left ventricular ejection fraction, *TAPSE* tricuspid annular plane systolic excursion, *eGFR* estimated glomerular filtration rate, *HFrEF* heart failure with reduced ejection fraction, *HFmrEF* heart failure with mildly reduced ejection fraction, *HFpEF* heart failure with preserved ejection fraction, *BB* beta blockers, *ACEi* angiotensin converting enzyme, *ARBs* angiotensin receptor blockers, *ARNI* angiotensin receptor/neprilysin inhibitor, *MRAs* mineralocorticoid receptor antagonists, *STEMI* ST-elevation myocardial infarction, *NSTEMI* non-ST elevation myocardial infarction, *UA* unstable angina

**Table 4:** Occurrence of main adverse outcomes in the ACS total population and according to SGLT2i introduction

Outcomes	ACS (*N *= 69)	SGLT2i on therapy (*N* = 32)	SGLT2i off therapy (*N* = 37)	*p*-value
CV death/HFH, n (%)	7 (10)	0 (0)	7 (18.9)	0.012
CV death, n (%)	6 (8.7)	0 (0)	6 (16)	0.026
HFH, n (%)	2 (3)	0 (0)	2 (5.4)	0.49
All-cause death, n (%)	8 (11.5)	0 (0)	8 (21.6)	0.005

*CV *cardiovascular,* HFH *heart failure hospitalization,* ACS *acute coronary syndrome,* SGLT2i *sodium glucose cotransporter 2 inhibitors

**Fig. 3:**
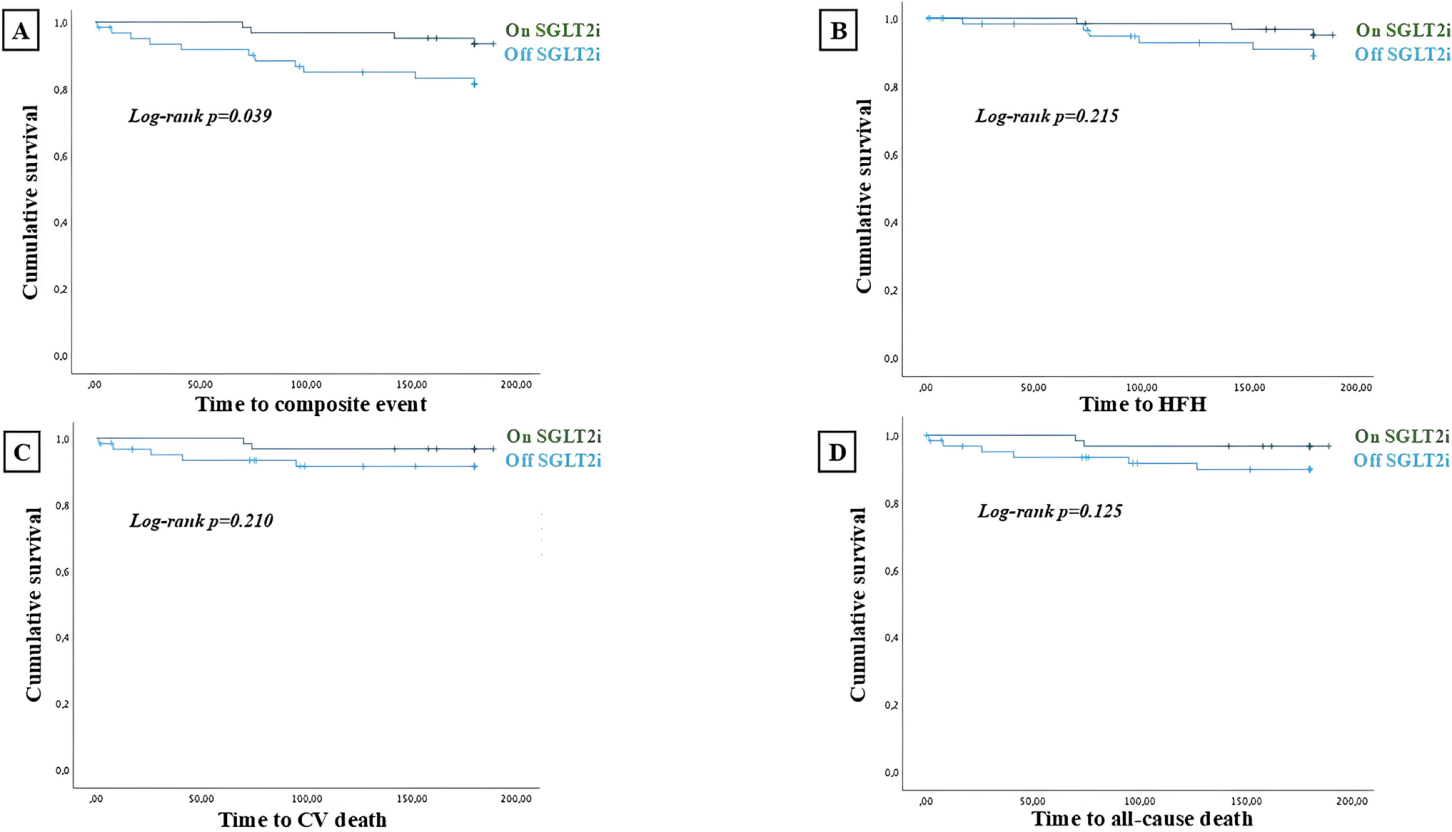
Survival analysis regarding the composite of CV death and HFH (**A**), each of the outcome of the composite endpoint (**B** and **C**) and all-cause death (**D**), in patients with de novo HF due to ischemic cardiomyopathy. Time to the event has been expressed in days. “on SGLT2i” defines patients who introduced SGLT2i during hospitalization while “off SGLT2i” defines patients who did not start SGLT2i during hospitalization *CV: cardiovascular; HFH: heart failure hospitalization; SGLT2i: sodium glucose cotransporter 2 inhibitors*

A further subgroup analysis has been performed regarding the patients with de novo HF. Baseline features and discharge therapy of this subpopulation has been represented in Table 5. Occurrence of main adverse outcomes in this subpopulation has been represented in Table 6. The Kaplan Meier survival analysis showed that patients with de novo HF who introduced SGLT2i during hospitalization experienced a significant reduced rate of the composite CV death/HFH (log-rank *p* = 0.039), but not CV death (log-rank *p* = 0.210), HFH (log-rank *p* = 0.215) and all-cause death (log-rank *p* = 0.125) (Fig. 3).

**Table 5:** Baseline features and discharge therapy of de novo HF subpopulation according to SGLT2i therapy

Variables	De novo HF (*N* = 126)	SGLT2i on therapy (*N* = 62)	SGLT2i off therapy (*N* = 64)	*p*-value
Age (± SD)	71 (**± **17)	71.5 (**± **14)	71 (**± **15)	0.798
Male sex, n (%)	92 (73)	45 (72.6)	47 (73.4)	0.914
ACS, n (%)	37 (29.3)	16 (25.8)	21 (32.8)	0.388
SAH, n (%)	100 (79.4)	50 (80.6)	50 (78)	0.727
Type II diabetes mellitus, n (%)	52 (41.2)	24 (38.7)	28 (43.7)	0.566
Dyslipidemia, n (%)	85 (67.4)	43 (69.3)	42 (65.6)	0.655
Family history of CVD, n (%)	31 (24.6)	16 (25.8)	15 (23.4)	0.758
COPD, n (%)	31 (24.6)	13 (21)	18 (28)	0.351
Smoking habit, n (%)	72 (57.1)	35 (65.5)	37 (57.8)	0.877
LVEF, % (± SD)	35 (**± **15)	32 (**± **16)	35 (**± **11.5)	0.215
TAPSE, mm (SD)	18 (5)	18 (6)	19 (5)	0.889
eGFR, ml/min (± SD)	66 (**± **37)	68 (**± **35)	63 (**± **41)	0.217
BB, n (%)	114 (90.5)	54 (87)	60 (93.7)	0.203
ACE-i/ARBs, n (%)	42 (33.3)	18 (29)	24 (29.7)	0.348
ARNI, n (%)	66 (52.4)	42 (67.7)	24 (37.5)	< 0.001
MRAs, n (%)	72 (57.1)	29 (46.7)	43 (67)	0.420
Loop diuretics, n (%)	86 (68.2)	43 (69.3)	43 (67)	0.794

*ACS* acute coronary syndrome, *SAH* systemic arterial hypertension, *CVD* cardiovascular disease, *COPD* chronic obstructive pulmonary disease, *LVEF* left ventricular ejection fraction, *TAPSE* tricuspid annular plane systolic excursion, *eGFR* estimated glomerular filtration rate, *BB* beta blockers, *ACEi* angiotensin converting enzyme, *ARBs* angiotensin receptor blockers, *ARNI* angiotensin receptor/neprilysin inhibitor, *MRAs* mineralocorticoid receptor antagonists, *SGLT2i* sodium glucose cotransporter 2 inhibitors

**Table 6:** Occurrence of main adverse events in the de novo HF subpopulation

Outcomes	De novo HF (*N* = 126)	SGLT2i on therapy (*N* = 62)	SGLT2i off therapy (*N *= 64)	*p*-value
CV death/HFH, n (%)	15 (11.9)	4 (6.4)	11 (17.2)	0.05
CV death, n (%)	7 (5.5)	2 (3.2)	5 (7.8)	0.439
HFH, n (%)	9 (7.1)	3 (4.8)	6 (9.3)	0.491
All-cause death, n (%)	8 (6.3)	2 (3.2)	6 (9.3)	0.274

*CV* cardiovascular,* HFH *heart failure hospitalization,* HF *heart failure,* SGLT2i *sodium glucose cotransporter 2 inhibitors

## Discussion

The spectrum of HF related to IHD is heterogeneous, therefore individualized therapeutic approaches are recommended, according to differences among patients and countries in terms of HF etiology, outcomes and management resources [24, 25]. Various well-established pharmacological treatments that have demonstrated survival benefits are accessible for patients with HF, such as β-blockers, ACE inhibitors/angiotensin receptor neprilysin inhibitor (ACEi/ARNI), angiotensin receptor blockers (ARBs), mineralocorticoid receptor antagonists (MRAs) and SGLT2i [1, 10]. Evidence regarding SGLT2i supports their uses across the LVEF spectrum and early after the acute phases of HF [10, 15, 16]. Their uses in the context of IHD is of interest considering the several pathophysiological and molecular aspects (i.e. atherosclerotic process, coronary microvascular dysfunction, ischemia–reperfusion injury and oxidative stress) involved in the ischemic heart both during the acute phases following myocardial ischemia and in the long-term remodeling processes [26, 27].

Currently, the effect of early SGLT2i use in IHD, in particular following ACS, is debated and many studies reported ambiguous results [17–23]. Zelniker et al. [28] demonstrated that SGLT2i has moderate benefits on atherosclerotic related major adverse CV events, in patients with established atherosclerotic CV disease, as well as a benefit on reducing HFH and progression of renal disease regardless of existing atherosclerotic CV disease or history of HF [28]. Furthermore, the effects of Empagliflozin on reducing CV death in patients with atherosclerotic CV diseases have been more compared to Canagliflozin and Dapagliflozin. The EMPAREG-OUTCOME trial [29] demonstrated that Empagliflozin reduced CV, all-cause death and hospitalization for HFH over placebo in 7.020 patients with peripheral, coronary and cerebrovascular atherosclerotic disease and type 2 diabetes, demonstrating its benefit in the population of ischemic patients.

SGLT2i have been studied also in the acute setting of HF, but trials regarding IHD are still lacking. In The EMMY trial [30] analyzed the effects of Empagliflozin within 72 h after percutaneous coronary intervention in the setting of a large myocardial infarct. Empagliflozin significantly reduced NT-proBNP levels compared to placebo by 15%. Also, LVEF improved significantly by almost 1.5%, mean *E*/*e*′ reduction was 6.8%, LV end-systolic volume (LVESV) was lowered by 7.5 mL and LV end-diastolic volume (LVEDV) was lowered by 9.7 mL. The EMPULSE trial [15] tested the early administration of Empagliflozin in patients with acute HF after clinical stabilization for up to 90 days. The primary composite endpoint of all-cause death, number of HF events, time to first HF event and ≥ 5 point difference in change from baseline in the Kansas City Cardiomyopathy Questionnaire at 90 days was achieved in more patients treated with Empagliflozin, regardless of diabetes status and LVEF. The EMPACT-MI trial [17] did not show a reduced risk of all-cause mortality and HFH in patients at risk for HF and recent ACS. Chen et al. [31] demonstrated the CV protective effect of the early initiation of SGLT2i in patients with a known history of type 2 diabetes and new-onset ACS. The study involved a total of 232 patients hospitalized for ACS, of whom 45.3% had ST elevation myocardial infarction (STEMI). The early initiation of SGLT2i therapy was associated with a reduced risk of rehospitalization for HF and a higher rate of control over angina symptoms. Notably, only 1.7% of patients had a history of previous HF prior to hospital admission. However, SGLT2i did not show a significant association with improved in-hospital clinical outcomes, such as CV mortality, adverse CV outcomes or composite rehospitalization rates for ACS or HF. Similarly, Liu et al. [32] confirmed the CV efficacy of SGLT2i post-ACS in 925 Chinese patients with type 2 diabetes and a new-onset ACS. The treatment group received SGLT2i during ACS hospitalization and continued therapies throughout the follow-up period. After one year, patients treated with SGLT2i therapy showed a significantly lower cumulative incidence of adverse events, CV death and HFH across various subgroups. Sinha et al. [21] showed a significant reduction in the risk of adverse events, all-cause mortality, CV mortality and CV-related hospitalizations among patients receiving SGLT2i. A trend towards a reduced risk of recurrent ACS has been observed, although the difference did not reach statistical significance. Kanaoka et al. [23] investigated the efficacy of SGLT2i in real-world patients with new-onset ACS and demonstrated a lower risk of composite all-cause mortality and HF related ACS rehospitalization in 115.612 patients presenting with ACS and severe HF. This effect was not observed in patients with ACS but without severe HF. The SGLT2i use was associated with lower all-cause mortality and HF rehospitalization but did not influence ACS rehospitalization. This study suggests the potential benefit of early SGLT2i use in patients with new-onset ACS complicated by severe HF. Mao et al. [33] demonstrated a statistically significant reduction in the cumulative risk of HFH in diabetic patients with ACS treated with Dapagliflozin, both during hospitalization and after discharge. Recently, Maremmani et al. [34] demonstrated a beneficial effect of both early and delayed SGLT2i initiation in patients with AMI in terms of all-cause mortality regardless of HF presence [34].

Despite the substantial evidence supporting the protective effects of SGLT2i on atherosclerotic CV disease, in a recent meta-analysis, Tsai et al. [35] found no reduction in the risk of ACS in patients chronically taking SGLT2i, although SGLT2i use was associated with a reduced risk of CV mortality and all-cause mortality. This recent meta-analysis shows that the use of SGLT2i did not significantly change the incidence of ACS.

In this context, patients who early started SGLT2i, before hospital discharge, experienced a significant lower rate of CV death/HFH (log-rank *p* = 0.002), all-cause death (log-rank *p* = 0.038), CV death (log-rank *p* = 0.038) and HFH (log-rank *p* = 0.032) at 6 months follow up (Fig. 1). Among patients admitted with HF and ACS at admission, those who early started SGLT2i during hospitalization experienced a significant lower rate of CV death/HFH (log-rank *p* = 0.008), all-cause death (log-rank *p* = 0.005), CV death (log-rank *p* = 0.015), but not HFH (log-rank *p* = 0.155) at 6 months follow up (Fig. 2). Among patients of the subpopulation with de novo HF, those who introduced SGLT2i during hospitalization experienced a significant lower rate of the composite of CV death/HFH (log-rank *p* = 0.039) (Fig. 3).

Moreover, in the general population, patients receiving SGLT2i early on during hospitalization were also significantly more treated with an ARNI at discharge (p < 0.001), hypothesizing a favorable effect driven by early use of SGLT2i on ARNI tolerance in these patients. This aspect may be of particular interest given the amplified beneficial effect of early initiation of comprehensive HF therapy. The pathophysiological mechanisms underlying the positive effects of SGLT2i in patients with HF following ACS may be linked to the effect of SGLT2i on myocardial remodeling occurring within the heart after AMI, as well as myocardial metabolism.

Clinical effects of SGLT2i on HF are linked to the ability of reversing cardiac remodeling and modify cardiac energetics [36–43]. It is reasonable to hypothesize that the early beneficial effects of SGLT2i in patients with ischemic HF and following ACS, may be also related to reduction of ventricular volume load, inflammation, oxidative stress, as well as microvascular function and myocardial energetic improvement [40].

The results of our study may highlight the potential benefits of early initiation of SGLT2i in patients with ischemic cardiomyopathy, and potentially also in those with recent ACS and de novo HF, where early use of SGLT2i remains a topic of debate. Our findings may offer a perspective more grounded in the etiological and pathophysiological mechanisms underlying HF, irrespective of LVEF or clinical presentation. Given the cardiometabolic and cardiorenal effects of SGLT2i, future studies on this topic may also focus on alternative outcomes, such as biomarkers and advanced imaging parameters, rather than only short-term clinical endpoints, considering the long-term structural changes that occur in the ischemic heart.

Our manuscript has several limitations. The results should be confirmed by a larger population and a longer follow up should be required. The analysis has been performed on the main outcomes for HF and the analysis in terms of rehospitalization due to IHD, revascularization and reinfarction is missing. In our ACS population, patients with a history of HF prior index hospitalization have been also included in the final analysis. An in-depth analysis regarding safety endpoints is missing.

## Conclusion

HF is a main socioeconomic problem worldwide [1, 2]. IHD represents the main cause of HF, including both ACS and chronic coronary syndrome [1, 2, 5]. The introduction of HF disease modifiers (i.e. BB, SGLT2i, ACEi/ARNI and MRAs) changed the course of the disease. Recent Guidelines [1, 10] and main randomized controlled trials [13–16] demonstrated the role of comprehensive therapy in reducing the rate of adverse events in HF patients. In particular, during the last years, SGLT2i revolutionized the treatment of HF, regardless of LVEF, clinical presentation, diabetes mellitus and chronic kidney disease. Several studies [17–23, 31–35] showed contrasting evidence regarding their use in patients with IHD, in particular with recent ACS. In this manuscript we wanted to address this gap, focusing on the early, in-hospital use of SGLT2i in the ischemic HF population, the most represented in western countries, demonstrating a favorable effect of this drug class in terms of a composite of CV death/HFH, all-cause death, CV death and HFH, at 6 months follow up. The same treatment strategy based on SGLT2i, has been associated with a reduced rate of CV death/HFH, all-cause death, CV death, but not HFH, in the ACS population, as well as in the de novo HF subpopulation, in term of the composite of CV death/HFH.

Several pathophysiological hypotheses may be related to these results, in particular the effect of SGLT2i on LV remodeling processes, myocardial energetic, cardiometabolic aspects, coronary plaque stabilization [36–46]. Several studies in this direction are currently ongoing [44, 45]. In this regard, taking into account HF aetiology and management strategies [25], as well as stratifying HF patients based on their underlying pathophysiological profile [47–51], this study may serve as a valuable starting point for future research aligned with the rationale of an even more personalized and tailored approach to HF.

## Data Availability

The data that support the findings of this study are available from the corresponding author upon reasonable request.
